# Auditory and motion metaphors have different scalp distributions: an ERP study

**DOI:** 10.3389/fnhum.2015.00126

**Published:** 2015-03-13

**Authors:** Gwenda L. Schmidt-Snoek, Ashley R. Drew, Elizabeth C. Barile, Stephen J. Agauas

**Affiliations:** Department of Psychology, Hope CollegeHolland, MI, USA

**Keywords:** metaphor, motion, auditory, familiarity, imageability, embodied language, N400, P600

## Abstract

While many links have been established between sensory-motor words used literally (kick the ball) and sensory-motor regions of the brain, it is less clear whether metaphorically used words (kick the habit) also show such signs of “embodiment.” Additionally, not much is known about the timing or nature of the connection between language and sensory-motor neural processing. We used stimuli divided into three figurativeness conditions—literal, metaphor, and anomalous—and two modality conditions—auditory (*Her limousine was a privileged snort*) and motion (*The editorial was a brass-knuckle punch*). The conditions were matched on a large number of potentially confounding factors including cloze probability. The electroencephalographic response to the final word of each sentence was measured at 64 electrode sites on the scalp of 22 participants and event-related potentials (ERPs) calculated. Analysis revealed greater amplitudes for metaphorical than literal sentences in both 350–500 ms and 500–650 ms timeframes. Results supported the possibility of different neural substrates for motion and auditory sentences. Greater differences for motion sentences were seen in the left posterior and left central electrode sites than elsewhere on the scalp. These findings are consistent with a sensory-motor neural categorization of language and with the integration of modal and amodal information during the N400 and P600 timeframes.

## Introduction

Now that many neural links have been established between language and action (e.g., Wallentin et al., 2005) it is time to move beyond the debate about whether language and cognition are embodied and to begin investigating the timing and nature of the neural link between language and sensory-motor aspects of experience (Chatterjee, 2010). Many investigators have demonstrated links between literally used action words (grasp the ball) and action areas of the brain, but fewer have done so with action words used metaphorically (grasp the idea), which do not literally refer to actions. In the current study we investigate the metaphorical use of motion and auditory words using event-related potentials (ERPs). Using this method allows us to examine the timing of the link between conceptual and sensorimotor aspects of a semantic concept.

### Modality and Metaphor

Most psychology and cognitive science researchers have previously regarded language and cognition as amodal; however recently the notion that cognition may be grounded in sensory-motor experience and embodied has become dominant (Barsalou, 2008). Several studies have shown the activation of sensory or motor regions of the brain during the processing of words or other stimuli depicting actions or sensory experiences (Pulvermüller, 2005; Wallentin et al., 2005; Daselaar et al., 2010). Nevertheless intense debate surrounds the embodied view of language (Gibbs, 2013a). To move forward, it is not necessary to continue to demonstrate links between cognition and action. Rather, work must now focus on the nature of that embodiment, the direction of influence between modal and amodal representations, and the timing of the connection between them (Mahon and Caramazza, 2008; Chatterjee, 2010; Gibbs, 2013a). For example, Rueschemeyer et al. (2010) found participants performing an intentional action (but not a non-intentional action) showed a priming effect for processing words depicting manipulable compared to nonmanipulable objects. If something like intentionality is important in showing a link between sensorimotor processes and language, the nature of embodiment may be more complex than previously thought.

Support for embodied theories of language has frequently come from reports of activations in sensory-motor areas of the brain triggered by action words (e.g., Barsalou, 1999; but see Mahon and Caramazza, 2008, who also discuss other interpretations of these findings). Using metaphor is a particularly compelling way to examine embodied theories of language. Showing the neural activation associated with words referring to physical actions (*grasp the ball*) does not go as far as extending this to action words used metaphorically (*grasp the idea*). When a word with sensory-motor properties used in a non-literal way recruits the sensory-motor regions of the brain, this activation provides strong support for a robust association between physical experience and completely abstract concepts in the brain, such as understanding (*grasping*) an idea. While some studies failed to find this association (e.g., Aziz-Zadeh and Damasio, 2008; Cardillo et al., 2012), some recent attempts have been successful (e.g., Cacciari et al., 2011; Desai et al., 2011, 2013). Most studies have reported links between action concepts and the motor system, but links in other modalities such as texture and the sensory system (e.g., Lacey et al., 2012) have also been reported. We extend this work by comparing metaphors based on two modalities, auditory (*The flowers were a colorful clamor*) and motion (*Her inquiries were a nervous scamper*).

Identifying when and how modal and amodal representations interact in the brain is important for understanding the nature of embodiment. For example, the link between the amodal and modal representation of a specific word or concept may only happen at a specific stage of processing rather than globally (Ritchie, 2008). Neuroimaging studies by their nature do not provide precise timing information, but electrophysiological methods do. In particular, the N400 ERP component, a negativity occurring about 400 ms after stimulus presentation, is sensitive to anomaly as in *He took a sip from the transmitter*. A larger N400 amplitude has traditionally been considered an index of the ease of semantic integration (Kutas and Hillyard, 1980). Current thinking suggests the N400 is more specifically associated with neural access to initial conceptual representations or semantic retrieval (Van Petten and Luka, 2006). In fact, Federmeier and Laszlo (2009) proposed the N400 is associated with the binding of data from various modalities, creating a multimodal conceptual representation that is dynamically created and highly context dependent (Kutas and Federmeier, 2011). The N400 is an ideal measure for investigating the timing of the neural basis of metaphor based on different modalities and is our primary dependent measure.

### The Current Study

Stimuli in the current study were divided into three figurativeness conditions (literal, metaphor and anomalous) crossed with two modality conditions (auditory and motion). Sentences included auditory literal (*His comeback was a haughty **snort***), auditory metaphor (*Her limousine was a privileged **snort***), motion literal (*The blow was a single **punch***), motion metaphor (*The editorial was a brass-knuckle **punch***), and anomalous. See Table 1 for more examples. The conditions were matched on a large number of potentially confounding factors (Cardillo et al., 2010).

**Table 1:** **Examples of each sentence type**.

	Motion	Auditory
**Literal**	His move was a quick **dodge**.	The only noise was a **flush**.
	Her punishment was a strong **slap**.	The sound was her bitter **sob**.
**Metaphorical**	His smile was a charming **dodge**.	His memoirs were a toilet **flush**.
	The rejection letter was a **slap**.	Her marriage was a long **sob**.
**Anomalous**	The light bulb was a bright **dodge**.	The hard working ant was a diligent **flush**.
	The cat’s nine lives were an odd **slap**.	The flock of birds was a friendly **sob**.

The purpose of the current study was to use ERP to investigate the nature and time course of metaphor comprehension based on two different modalities. We compared the neural processing of motion and auditory modalities. It is common in ERP studies to use differences in component distribution across the scalp to infer differences in neural areas recruited. Kutas and Federmeier (2011) discuss a number of such examples with the N400. We hypothesize that if the N400 reflects the binding of data from different modal and amodal representations, different parts of the brain should be recruited in addition to language areas for each modality–for example the motor cortex for motion sentences, and the auditory cortex for auditory sentences. We predicted a difference in the scalp distribution of the N400 for the two modalities demonstrating different underlying patterns of activation at 400 ms post stimulus.

While examining our data in the present study, it became apparent that differences in positivity were occurring in the P600 time range. The P600 ERP component has traditionally been considered an index of syntactic error processing although it is now known to be involved in various complex sentences processing mechanisms (e.g., Gouvea et al., 2010; Kutas and Federmeier, 2011), including semantic integration (Brouwer et al., 2012). We thus added the P600 as an additional dependent measure.

## Materials and Methods

### Participants

Participants were 28 volunteers with at least 1 year of post-secondary education from the Hope College community. Data from two participants, who scored less than 60% correct in the anomalous condition, were excluded since their score suggests they may not have comprehended many metaphors. Data from an additional four participants were excluded due to insufficient acceptable trials (less than 20 per condition). The remaining 22 participants (17 women, mean age 20.8 years, range 18–23, mean years of education 14.5, range 13–16) were native English speakers, and had no history of neurological or psychiatric disorders. All participants were right-handed with a mean handedness score of 0.84 (*SD* = 0.16) (Annett, 1970); 11 reported left-handed family members. This study was approved by the Hope College Human Subjects Review Board and all participants provided written informed consent prior to participation.

### Creation of Stimuli

A preliminary list of 411 sentences was compiled consisting of literal, metaphorical, and anomalous sentences. Literal and metaphorical sentences were obtained from Cardillo et al. (2010). Cardillo et al. matched sentences on 10 dimensions: length, frequency, concreteness, familiarity, naturalness, imageability, figurativeness, interpretability, valence, and valence judgment reaction time. The sentences began with a subject followed by the past or present tense form of the verb “be” followed by an adjective for the object (e.g., His job was an endless). Each sentence ended with either an auditory or motion target word as the object (e.g., groan). Motion words were physical actions depicting motion such as climb, dig, and stampede, whereas auditory words included sounds like sneeze, chirp, and hiss. For each target word, a literal and a metaphorical sentence were written (Table 1). Thus, each target word was used both literally and figuratively based on the context of the noun phrase. In the present study, the same sentence structure and target words were used to create anomalous sentences. Anomalous sentences were created by the authors and had neither a literal nor metaphorical meaning. These sentences were included as a control condition for comparison with the literal and metaphorical conditions.

Before the final selection of stimuli, three preliminary studies further characterized the sentences. Fifty-two native English speakers, who did not participate in the main experiment, completed a cloze probability questionnaire by finishing each sentence with the first word that came to mind. Words were keyed into a spreadsheet using a standard computer keyboard. Excluding one participant due to non-compliance with task instructions, data from the remaining 51 participants (35 women, mean age 19 years) were used to calculate the cloze probability of each sentence. The sum of answers matching the actual target word was divided by the number of participants to measure the sentence ending predictability.

A second questionnaire was completed by 20 native English speakers (14 women, mean age 18 years) who did not participate in the main experiment. Using a 7-point scale (1 = low, 7 = high), each participant rated the familiarity and imageability of 277 sentences (70 literal, 70 metaphorical, 137 anomalous). Responses were keyed into a spreadsheet using a standard computer keyboard. The anomalous sentence ratings were added to the collection of literal and metaphorical sentence ratings.

Third, a pilot test was conducted to attain average response times and accuracy ratings for each sentence. Twenty native English speakers (13 women, mean age 18 years) were tested on the original stimulus set of 411 sentences using the procedure from the main experiment.

The resulting cloze probability, familiarity, imageability, pilot response time, and pilot accuracy ratings were used in the final selection of stimuli to create the most balanced stimuli possible. In addition, several other factors were balanced. Crucially, modality (auditory, motion) and figurativeness (literal, metaphorical) factors did not differ on cloze probability ratings (*p*s > 0.05).

Some of the stimuli had an adjective modifying the final target word (Cardillo, 2010). Across motion and auditory sentences, there was no difference in the number of sentences having an adjective modifying the object (target) and those that did not (*p* > 0.05). The frequency and concreteness of adjectives in motion vs. auditory sentences did not differ as a whole or looking at literal and metaphorical sentences separately (all *p*s > 0.05). However, several factors across figurativeness conditions for either modality could not be balanced (*p*s < 0.05). Table 2 lists all the factors considered and descriptive statistics for the four sentence types. Table 3 gives the results of *t*-tests conducted to assess differences. The final stimulus set contained 300 sentences, 50 in each condition.

**Table 2:** **Characteristics of the final stimuli**.

	Motion metaphor		Motion literal		Auditory metaphor		Auditory literal	
	*M*	*SD*	*M*	*SD*	*M*	*SD*	*M*	*SD*
**Sentence**
Cloze probability	0.01	0.03	0.02	0.04	0.01	0.02	0.02	0.07
Familiarity (1–7 scale)	4.03	1.02	5.19	1.02	3.84	1.23	5.24	0.74
Imageability (1–7 scale)	3.99	0.85	5.73	0.87	4.04	0.88	4.96	0.75
Figurativeness (1–7 scale)	5.47	0.75	2.20	0.80	5.35	0.68	2.30	0.71
Valence (% positive)	0.29	0.32	0.29	0.28	0.26	0.32	0.16	0.26
Valence RT (ms)	1498	188	1512	195	1485	271	1391	199
Pilot accuracy (%)	0.69	0.22	0.81	0.20	0.60	0.23	0.80	0.20
Pilot RT (ms)	1314	931	853	455	1446	771	996	830
# characters	31.0	3.8	30.4	4.1	31.0	4.4	29.7	4.2
# words	6.1	0.5	6.0	0.4	6.0	0.5	5.9	0.4
# content words	3.1	0.4	3.0	0.4	3.0	0.5	2.9	0.4
KF frequency	79	100	72	95	60	82	90	146
BN frequency	78	96	76	163	75	147	96	150
Concreteness (0–700 scale)	421	63	433	52	441	66	418	63
**Target word**
#characters	5.1	1.3	(same as motion metaphor)		5.5	1.6	(same as auditory metaphor)
KF frequency	10	32			18	38
BN frequency	10	21			28	68
Concreteness (100–700 scale)	451	52			457	62

*KF frequency = frequency value from Kučera and Francis (1967). BN frequency = SUBTLEX frequency value from Brysbaert and New (2009). Familiarity, Imageability and Valence reflect ratings of the entire sentence. Frequency and Concreteness ratings reflect the mean value of all content words in each sentence. Since concreteness ratings are based on published norms of individual words, they do not necessarily reflect the concreteness or imageability of the sentence as a whole. Valence ratings were binary; subjects rated sentences as positive or neutral/negative*.

**Table 3:** **Comparison of final stimuli across modality and figurativeness conditions**.

	Modality				Figurativeness			
	Motion metaphor/Auditory metaphor		Motion literal/Auditory literal		Motion literal/Motion metaphor		Auditory literal/Auditory metaphor	
	*t*	Sig.	*t*	Sig.	*t*	Sig.	*t*	Sig.
**Sentence**
Cloze probability		ns		ns		ns		ns
Familiarity		ns		ns	5.66	<0.001	6.91	<0.001
Imageability		ns	4.74	<0.001	10.13	<0.001	5.63	<0.001
Figurativeness		ns		ns	−20.95	<0.001	−21.98	<0.001
Valence		ns	2.4	0.02		ns		ns
Valence RT		ns	3.1	0.003		ns	1.99	0.05
Pilot accuracy	1.95	0.055		ns	2.92	0.004	4.59	<0.001
Pilot RT		ns		ns	−3.14	0.002	−2.81	0.006
# characters		ns		ns		ns		ns
# words		ns		ns		ns		ns
# content words		ns		ns		ns		ns
KF frequency		ns		ns		ns		ns
BN frequency		ns		ns		ns		ns
Concreteness		ns		ns		ns		ns
**Target word**
#characters		ns		ns		n/a		n/a
KF frequency		ns		ns		n/a		n/a
BN frequency		ns		ns		n/a		n/a
Concreteness		ns		ns		n/a		n/a

*Degrees of freedom = 98 for each t-test. ns = non-significant, p < 0.05 (two-tailed). See the legend for Table 2 for information about the items listed*.

### Procedure

Participants were tested individually in a single experimental session. Stimuli were presented using E-Prime software (Psychological Software Tools, Pittsburgh, PA, USA) in 20pt Arial bold font, with white text on a black background. During a practice block of 10 sentences, participants were acclimated to the task and given verbal feedback regarding their task performance and blinking. Each trial began with the beginning of the sentence (the entire sentence except the last word). Participants controlled the advancement of the trial by pressing the spacebar when ready. Next, an automatic timed sequence occurred in which participants were asked not to blink: fixation cross (500 ms), final word of the sentence (1200 ms), and a response screen (limited to 5000 ms). The response screen instructed participants to indicate whether the presented sentence was literal, metaphorical, or anomalous via keyboard response with the first three fingers of the right hand. This ensured that metaphorical trials were processed as metaphorical by the participant since incorrect trials were discarded. It also ensured the subjects were attending to and processing the sentences. However it may be that a certain neural pattern motivated the participants to give a particular behavior response, triggering our results and resulting in circular reasoning. The present results must be interpreted with this caveat in mind. Once an answer was given, the next trial began after a randomly assigned intertrial interval between 900 ms and 1150 ms in 50 ms increments.

Each of the 17 blocks contained an equal number of each sentence type in a unique random order for each participant. An additional version of the experiment was formed by reversing the order of the blocks. Participants were randomly assigned to one of the two block orders to reduce word priming effects in the experiment. Participants controlled their resting time upon the completion of every block. The total duration of the study was approximately two hours.

### Electrophysiological Recording

Scalp activity was recorded with a 64 channel BioSemi ActiveTwo system (BioSemi Inc., Amsterdam, Netherlands) with an analog-to-digit rate of 512 Hz and a bandwidth of 104 Hz. A Common Mode Sense (CMS) active electrode was used as the reference, and a Driven Right Leg (DRL) passive electrode was used as the ground. Active Ag-AgCl pin-type electrodes were inserted into a Lycra head cap with locations based upon the American Electroencephalographic Society (1994). Electrooculograms (EOG) were recorded using flat-type electrodes placed on the left and right infraorbital ridge and outer cantus. In addition, two more flat-type electrodes were placed on the left and right mastoids. Individual electrode offsets were kept between ±30 mV.

**Figure 1:**
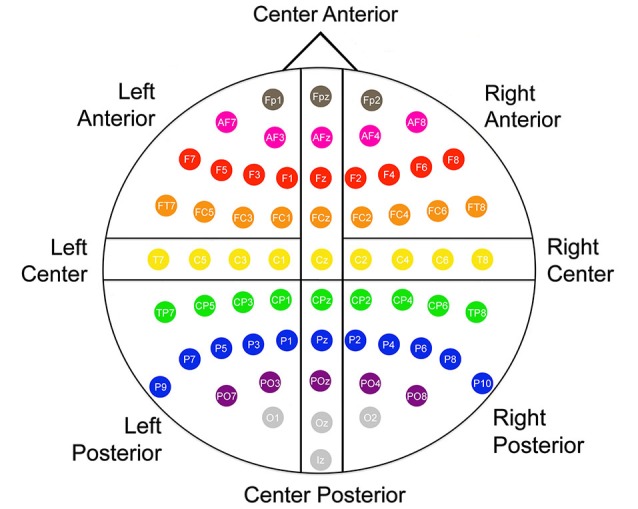
**Montage and scalp region designations used in all analyses**.

Offline, electroencephalography (EEG)/ERP analyses were conducted using EMSE Suite software (Source Signal Imaging Inc., San Diego, CA, USA). The left and right mastoid recordings were averaged and used as the offline reference. A digital bandpass filter of 0.01–30 Hz was applied to the EEG recordings, and then an individual eye artifact filter removed eye movements for each participant. ERPs were obtained through stimulus-locked averaging of each condition with an epoch extending from 200 ms pre-stimulus to 800 ms post-stimulus. Trials in which EEG or EOG channels exceeded ±50 µV, or in which the participant did not respond correctly in 5000 ms were eliminated. The remaining segments were baseline corrected and then averaged to create ERP waveforms for each participant. The mean number of trials averaged per condition per participant across all cells of data was 35.6 (*SD* = 6.9, range 20–50). Across the six conditions, the condition with the smallest number of mean trials per participant per condition was the auditory metaphor condition at 30.0 (*SD* = 6.4) and the condition with the largest number was the auditory anomalous condition with 40.0 (*SD* = 7.1). Figure 1 shows how the 64 electrode sites were divided into the following eight scalp regions: Left Anterior (FP1, AF7, AF3, F7, F5, F3, F1, FT7, TCF, FC3, FC1), Left Center (T7, C5, C3, C1), Left Posterior (TP7, CP5, CP3, CP1, P9, P7, P5, P3, P1, PO7, PO3, O1) Center Anterior (FPz, AFz, Fz, FCz, Cz), Center Posterior (CPz, Pz, POz, Oz, Iz), Right Anterior (FP2, AF4, AF8, F2, F4, F6, F8, FC2, FC4, FC6, FT8), Right Center (C2, C4, C6, T8), Right Posterior (CP2, CP4, CP6, TP8, P2, P4, P6, P8, P10, PO4, PO8, O2). We operationalized the N400 amplitude as the area under the curve from 350 ms to 500 ms and the P600 amplitude as the area under the curve from 500 ms to 650 ms, based on visual inspection of grand averages.

## Results

### Behavioral

The mean accuracy score across participants was 0.76 (*SD* = 0.05) and only correct trials were included in the ERP and reaction time analyses. A 2 (modality) × 3 (figurativeness) repeated measures ANOVA using the Huynh-Feldt sphericity correction was conducted on mean accuracy scores revealing an effect of figurativeness, *F*_(2,42)_ = 8.2, *p* = 0.003, *ε* = 0.784 and a modality × figurativeness interaction, *F*_(2,42)_ = 6.1, *p* = 0.005, *ε* = 1.0 (Degrees of freedom are reported with sphericity assumed throughout this manuscript). The interaction can be explained by an effect of modality for metaphors, *F*_(1,21)_ = 15.8, *p* = 0.001, but not for literal or anomalous sentences, *p*s > 0.05. Planned comparisons revealed that metaphorical sentences (*M* = 0.68, *SD* = 0.13) were processed less accurately than either literal (*M* = 0.78, *SD* = 0.09, *t*_(21)_ = 3.0, *p* = 0.006) or anomalous (*M* = 0.83, *SD* = 0.11, *t*_(21)_ = 3.2, *p* = 0.005) sentences.

A 2 (modality) × 3 (figurativeness) repeated measures ANOVA using the Huynh-Feldt sphericity correction was conducted on mean reaction times revealing only an effect of figurativeness, *F*_(2,42)_ = 8.3, *p* = 0.001, *ε* = 0.98. Planned comparisons revealed that metaphorical sentences (*M* = 855 ms, *SD* = 201 ms) were processed more slowly than either literal (*M* = 772 ms, *SD* = 245 ms, *t*_(21)_ = 2.7, *p* = 0.01) or anomalous (*M* = 705 ms, *SD* = 182 ms, *t*_(21)_ = 3.9, *p* = 0.001) sentences.

### Electrophysiological

#### N400

A 2 (modality) × 3 (literal/metaphor/anomalous) × 8 (scalp region) repeated measures ANOVA using the Huynh-Feldt sphericity correction was conducted to assess differences in N400 amplitude. This revealed a main effect of figurativeness, *F*_(2,42)_ = 24.0, *p* > 0.001, *ε* = 0.79, a main effect of scalp region, *F*_(7,147)_ = 3.6, *p* = 0.025, *ε* = 0.37, and a trending modality × scalp region interaction, *F*_(7,147)_ = 2.5, *p* = 0.063, *ε* = 0.46.

**Figure 2:**
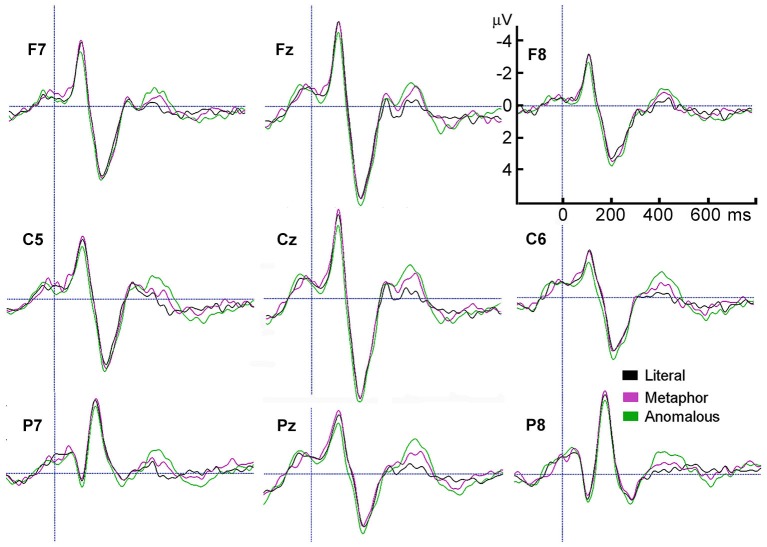
**Grand average ERPs of the last word in literal, metaphorical, and anomalous sentences, from representative electrodes in each of the 8 scalp regions plus Cz**.

**Figure 3:**
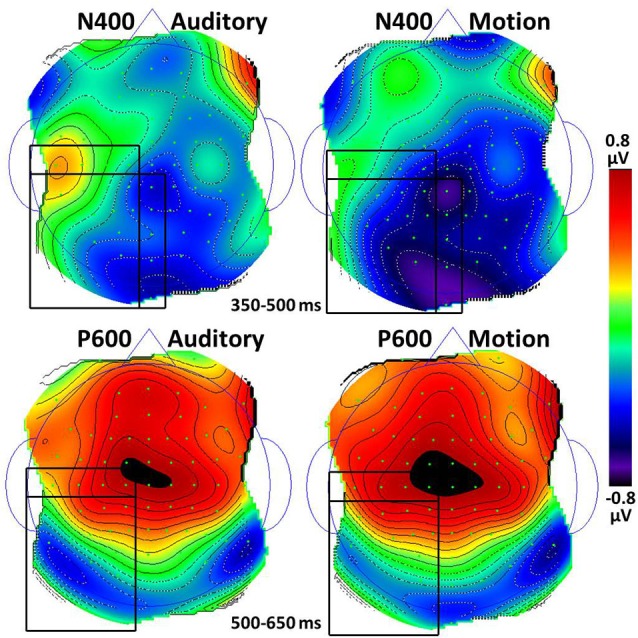
**Topographic maps of the N400 and P600 responses to the last word in auditory and motion sentences (anomalous excluded)**. For the N400, black rectangles indicate the Left Center, Left Posterior, and Center Posterior regions which had statistical differences between auditory and motion conditions, *p*s < 0.05. For the P600, black rectangles indicate the Left Center and Left Posterior regions which had differences approaching significance (*p*s < 0.18); the location × condition interaction was significant.

Figure 2 shows the largest N400 amplitudes were for anomalous sentences, followed by metaphorical sentences, *t*_(21)_ = 3.9, *p* = 0.001, followed by literal sentences, *t*_(21)_ = 4.8, *p* < 0.001. The modality × scalp region interaction reflects larger N400 amplitudes for motion than auditory sentences, with significant differences in the Left Center, *t*_(21)_ = 2.1, *p* = 0.047, Left Posterior, *t*_(21)_ = 2.5, *p* = 0.02, and Center Posterior, *t*_(21)_ = 2.3, *p* = 0.03 scalp regions (Figure 3).

To determine whether the figurativeness effect included a difference between literal and metaphorical sentences, the analysis was repeated without the anomalous condition, revealing a similar pattern with a main effect of figurativeness, *F*_(1,21)_ = 18.0, *p* < 0.001, a main effect of scalp region, *F*_(7,140)_ = 2.8, *p* = 0.055, *ε* = 0.37, and a modality × scalp region interaction, *F*_(7,147)_ = 2.7, *p* = 0.049, *ε* = 0.47. No other effects or interactions in either analysis were observed, *p*s > 0.05.

#### P600

Visual inspection of the findings suggested possible effects in the 500—650 ms time window, which we called the P600. To investigate this possibility, the same two analyses were conducted for the P600 amplitude. The first analysis revealed a main effect of figurativeness, *F*_(2,42)_ = 4.9, *p* = 0.012, *ε* = 1.0 (see Figure 3), a main effect of scalp region, *F*_(7,147)_ = 9.7, *p* < 0.001, *ε* = 0.47, and a modality × scalp region interaction, *F*_(7,147)_ = 3.5, *p* = 0.024, *ε* = 0.38, with no other effects or interactions, *p*s > 0.05. Figure 2 shows that anomalous sentences had a larger P600 amplitude than metaphorical sentences, *t*_(21)_ = 2.2, *p* = 0.04, but metaphor sentences did not differ from literal sentences, *p* > 0.28.

With the anomalous condition removed, only an effect of scalp region was found, *F*_(7,147)_ = 9.6, *p* < 0.001, *ε* = 0.43. Paired sample *t*-tests revealed no differences between auditory and motion sentences at any of the eight scalp regions, *t*s > 0.13. Similar to the N400 pattern, the modality × scalp region interaction reflects possibly larger P600 amplitudes for motion than auditory sentences in the Left Center and Left Posterior scalp regions, with few differences elsewhere (Figure 3).

#### Confounding Factors

The main effect of figurativeness for both the N400 and P600 may be confounded by the familiarity or imageability of the sentences. To explore this possibility, we created two levels of familiarity and imageability by performing a median split on the previously normed ratings. Separate 2 (high, low) × 8 (scalp region) ANOVA analyses using the Huynh-Feldt sphericity correction revealed significant effects for both the N400 (Familiarity, *F*_(1,21)_ = 33.3, *p* < 0.001; Imageability, *F*_(1.21)_ = 15.2, *p* = 0.001) and P600 (Familiarity, *F*_(1,21)_ = 5.8, *p* = 0.03; Imageability, *F*_(1.21)_ = 4.9, *p* = 0.04).

## Discussion

The current study explored the effect of modality on metaphor processing. We used ERPs to compare the processing of motion (*The partnership was a financial tailspin*) and auditory (*His emails were an insistent knock*) unfamiliar metaphors to literal and anomalous sentences using the same final word. We hypothesized a difference in the neural basis of motion compared to auditory metaphors. As predicted, we found a modality by scalp region interaction for the N400, and we discovered the same interaction for the P600. There were no interactions with figurativeness. These results support embodied views of language and suggests that metaphorical language is not qualitatively distinct from language in general. They also support the view that integration of modality and language information may be taking place in the 400 ms timeframe and later.

### Modality

This study suggests different neural processing of auditory and motion-based literal and metaphorical language for the N400 timeframe and also for the later P600 timeframe. Both components index various aspects of language processing. The N400 response to language stimuli represents aspects of semantic processing, including the possible building of a multimodal conceptual representation. The P600 is thought to underlie a revision process that occurs as more information is accounted for during the process of sentence comprehension (Kutas and Federmeier, 2011). Sensory-motor aspects of meaning may be accessed as early as 200 ms (Boulenger et al., 2012). The present findings suggest modality information is still processed and integrated in the 350–650 ms time window with two processes represented by the N400 and P600.

Many behavioral studies have demonstrated a link between the metaphorical use of language and sensory or motor processes, including novel sensory metaphors (*the past is heavy*) (e.g., Slepian and Ambady, 2014), conventional sensory metaphors (*anger is heat*) (Wilkowski et al., 2009), or conventional motion metaphors (*love is a journey*) (Gibbs, 2013b). Sensory motor regions of the brain have recently been shown to be activated in response to not only sensory-motor words but to those words used metaphorically (e.g., Cacciari et al., 2011; Lacey et al., 2012; Desai et al., 2013).

These studies link motor and language processing but do not provide information about the timing or nature of the link. Studies using EEG or MEG demonstrate activation of the motor cortex within 200 ms after the presentation of a word depicting action (Hauk and Pulvermüller, 2004). N400 effects have been found for the processing of visually perceived motion (Proverbio and Riva, 2009) and for the processing of a new meaning grounded in perception or action such as paddling a canoe with a Frisbee (Chwilla et al., 2007). The present findings extend these reports to literally and metaphorically used motion and auditory words presented in sentences. Our effects in the 350–650 ms timeframe suggest the integration and revision processes indexed by the N400 and P600 are likely to occur for both literal and metaphorical sentences with motion and auditory sensory-motor components in a later timeframe. Thus modality information continues to be processed during this time. This result is consistent with views that suggest the embodiment of language is not automatic and instant (Mahon and Caramazza, 2008; Rueschemeyer et al., 2010; Gibbs, 2013a) while not supporting an amodal view of language. (But see Mahon and Caramazza, 2008, who suggest that the activation of the literal meaning of metaphors during comprehension may be sufficient to modulate modality specific processes, although such process may not be required for comprehension). Since the effect existed for both literal and metaphorical sentences, metaphorical language may not be qualitatively distinct from language in general.

### Figurativeness

The current findings demonstrate a graded N400 effect with the amplitude of the N400 increasing from literal to metaphorical to anomalous sentences, consistently found across metaphor ERP studies (e.g., Arzouan et al., 2007; Lai et al., 2009). We also found a similar graded effect for the P600 in the 500–650 ms time range. Because our literal sentences were more imageable and familiar than our metaphorical sentences, it is probable that these factors can partially or completely account for our findings (Lee and Federmeier, 2008; Schmidt and Seger, 2009). Indeed, a median split based on these factors revealed significant differences in both the N400 and P600. The confounding by familiarity and imageability may need to be considered in comparisons between literal and metaphorical stimuli (Schmidt et al., 2010). ERP studies reporting a difference between literal and metaphorical stimuli, including ours, either do not mention matching familiarity between the sentences or if they do, do not balance the sentence types on familiarity. In these cases, metaphorical sentences are reported to be or appear to be less familiar than literal sentences. Indeed, when the metaphors are highly familiar or conventional, N400 differences between literal and metaphorical sentences are not always present (e.g., Balconi and Amenta, 2010). Studies reporting metaphor–literal differences in the N400 have also not addressed the imageability of the sentences used (Coulson and Van Petten, 2002, 2007; Kazmerski et al., 2003; Arzouan et al., 2007; Lai et al., 2009; Gold et al., 2010; Goldstein et al., 2012; Tzuyin Lai and Curran, 2013). Similarly, our metaphorical sentences were less imageable than our literal sentences.

## Conclusion

We report here the first ERP study of motion and auditory based metaphors. Our findings are consistent with the conclusion that the modality of the metaphor may influence its neural instantiation. The current findings also suggest that integration of modal and amodal meanings may be taking place during the N400 and P600 timeframes. Additional work is required to understand the exact nature of this integration. Further exploration of the interaction between the factor of modality on one hand and imageability and familiarity on the other hand is also warranted.

## Author Statement

GS, EB and SA were involved in the development of stimuli, initial design of the experiment and pilot testing. GS, AD, EB, and SA revised the study design based on pilot testing and finalized the selection of stimuli. AD and EB acquired the data. AD, EB and GS analyzed and interpreted the data. GS and AD wrote the first draft of the manuscript. GS, AD, EB, and SA contributed to and approved of the final manuscript.

## Conflict of Interest Statement

The authors declare that the research was conducted in the absence of any commercial or financial relationships that could be construed as a potential conflict of interest.
